# A Good Medical Practice of the Patients’ Right to Information: An Audit Cycle of Patient Understanding and Satisfaction With Information Provided to Patients With Acute Pancreatitis

**DOI:** 10.7759/cureus.47973

**Published:** 2023-10-30

**Authors:** Zohaib Jamal, Zeeshan Khawaja, Nowera Zafar, Muhammad Ijlal Haider, Naqqash Adnan, Asher Siddiqui, Imran Alam

**Affiliations:** 1 Department of Surgery, Wrightington, Wigan and Leigh NHS Foundation Trust, Wigan, GBR; 2 Department of General Surgery, East Lancashire Hospitals NHS Trust, Blackburn, GBR

**Keywords:** multi-organ failure, abdominal pain, morbidity and mortality, audit cycle, acute pancreatitis

## Abstract

Introduction

Acute pancreatitis (AP), characterized by the inflammation of the pancreas, is a common acute surgical condition accounting for approximately 3% of all surgical admissions with abdominal pain and has an incidence of approximately 56 cases per 100,000 population every year. The General Medical Council (GMC), National Institute for Health and Care Excellence (NICE), and Royal College of Nursing best practice guidelines recommend that such patients and their family members should be provided with both verbal and written information about acute pancreatitis and its management in a way that they can understand. The aim of this audit cycle was to find out the compliance with information provided to patients with acute pancreatitis as per the GMC good medical practice and NICE guidelines and assess their satisfaction.

Method

A closed-loop audit consisting of two cycles was carried out. Thirty patients who were admitted to the department of general surgery with acute pancreatitis were provided with a questionnaire containing 11 questions asking about the information provided to them about their condition by healthcare professionals; then, interventions were carried out in the form of developing patient information leaflets (PILs) and encouraging healthcare professionals to distribute them and provide information to the patients and their family members.

Results

Overall, improvements were seen in all aspects of the information being provided to patients, and particularly, more than 100% improvement was seen in patient satisfaction related to the information provided to them in the second cycle after the implementation of interventions.

Conclusions

This study concludes that patients should be given all the information they require in accordance with their right to information, in line with GMC best practice, NICE, and Royal College of Nursing best practice guidelines. A very effective way to improve the health outcomes and satisfaction of patients is to give them access to a patient information leaflet, which can allow patients to consider their options and understand what can happen during treatment, especially when doctors have limited time to carry out detailed discussions with the patient.

## Introduction

Acute pancreatitis (AP) is a common acute surgical condition and is characterized by the inflammation of the pancreas involving acinar cell injury along with both a local and systemic inflammatory response [1]. In the UK, it accounts for approximately 3% of all surgical admissions with abdominal pain and has an incidence of approximately 56 cases per 100,000 population every year [2,3]. It varies widely in terms of its severity from mild pancreatitis requiring conservative treatment only to severe pancreatitis with associated complications such as multi-organ failure and abdominal fluid collections, which can be associated with high morbidity and mortality [4,5]. The overall mortality of acute pancreatitis is approximately 5% in the UK but can be as high as 30% in severe acute pancreatitis [6]. Patients who develop an infected necrotic pancreatitis or organ failure are at the greatest risk of mortality among all etiologies of pancreatitis [7].

Patients with acute pancreatitis usually present with severe upper abdominal pain radiating to the back, nausea, and vomiting, and the diagnosis is established usually based on the following features: (a) sudden-onset persistent epigastric pain radiating to the back, (b) elevated lipase/amylase levels (>3 times the normal limit), and (c) characteristic radiological findings of contrast-enhanced CT scan and abdominal US scan (MRI can also be used but less commonly) [8]. Gallstones followed by excessive alcohol consumption account for 75% of instances of acute pancreatitis in the UK; however, alcohol intake is the most common cause worldwide [9].

The General Medical Council (GMC) best practice guidelines recommend that communication should be established with the patients in a way that helps them understand the information they need or want to know and advise to ensure that plans are made to address the patients' language and communication needs [10]. The National Institute for Health and Care Excellence (NICE) UK also advises to provide, as soon as possible after establishing the diagnosis, both verbal and written information, where applicable, to people with pancreatitis, as well as their family members or caregivers (where appropriate) [11]. According to the Royal College of Nursing, “person-centered care” indicates that the patient is an equal partner in the planning of their care and that their opinions are valued and respected [12].

We conducted an audit cycle in our hospital to find out compliance with information provided to patients with acute pancreatitis as per the GMC good medical practice and NICE guidelines and assess their satisfaction in terms of the information provided.

This article was previously presented as a meeting abstract at the Association of Surgeons in Training (ASiT) Surgical Conference 2023, in Liverpool, UK, and was published as a conference abstract in the British Journal of Surgery.

## Materials and methods

This audit cycle was conducted at the surgical department of Royal Albert Edward Infirmary, a district general hospital (DGH) based in the UK. The audit proposal was sent for approval and was registered with the research and audit department of Wrightington, Wigan and Leigh NHS Foundation Trust before commencing.

Thirty patients admitted with biochemical or radiological evidence of acute pancreatitis under the surgical team were included in the first cycle of the audit. All patients who presented with biliary colic, acute cholecystitis, choledocholithiasis, and malignant disease of the pancreas were excluded from the study. A self-structured questionnaire based on the NICE guidelines on the information and support section of acute pancreatitis in accordance with the GMC good medical practice guidelines was designed and provided to the patients included in the audit (Table 1). The purpose of conducting the audit was explained to the patients before administering the questionnaire, and written consent for their inclusion into the study was obtained. Patients were asked to mark their responses by answering either “Yes” or “No” to the questions in the questionnaire. The responses from all patients were recorded and analyzed using the Microsoft Excel software (Microsoft, Redmond, WA).

**Table 1:** Questionnaire designed based on the NICE guidelines on the information and support section of acute pancreatitis in accordance with the GMC good medical practice guidelines NICE: National Institute for Health and Care Excellence, GMC: General Medical Council

Number	(Please tick “Yes” or “No” based on the information provided to you and your understanding of the condition.)		
1	Are you aware of the reason for admission to the hospital?	Yes	No
2	Do you know/have you been explained what acute pancreatitis is?	Yes	No
3	Have you been explained what has/could have caused acute pancreatitis?	Yes	No
4	Have you been told about the investigations and their results used to confirm the diagnosis of acute pancreatitis?	Yes	No
5	Do you understand/have you been explained about the treatment of acute pancreatitis?	Yes	No
6	Have you been told about the potential complications of acute pancreatitis?	Yes	No
7	Have you been told that smoking and alcohol can cause pancreatitis?	Yes	No
8	Have you received enough verbal information about acute pancreatitis?	Yes	No
9	Have you received any written information about acute pancreatitis?	Yes	No
10	Did anyone make a diagram while explaining acute pancreatitis to you?	Yes	No
11	Were you satisfied with the information provided before this proforma to you?	Yes	No

**Figure 1:**
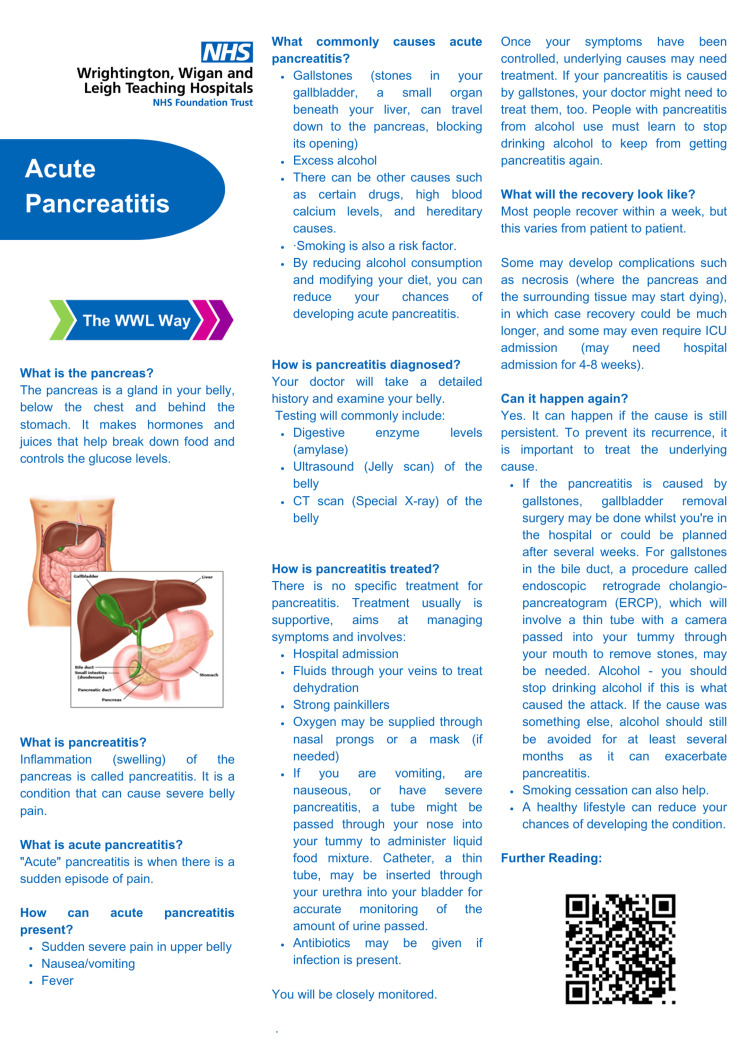
Acute pancreatitis patient information leaflet CT: computed tomography, ICU: intensive care unit, ERCP: endoscopic retrograde cholangiopancreatogram

The results were then discussed at the routine audit meeting, and interventions were implemented in the form of designing and distributing the patient information leaflet (PIL) on acute pancreatitis (Figure 1). The results of the first cycle of audit and proposed interventions were also communicated to the surgical doctors through emails, and PILs were printed and made available to all the ward clerks in surgical wards in our hospital. A second cycle was then conducted after two months on 30 more surgical patients to assess compliance with the good medical practice guidelines and satisfaction of the patients admitted with acute pancreatitis.

## Results

The first half of the audit cycle consisted of assessing the 30 questionnaires from patients admitted with acute pancreatitis, and the results showed only 40% patient satisfaction regarding the information provided. All patients admitted were aware of the reason for their admission in the hospital and the causes of acute pancreatitis, and 93% were aware of what acute pancreatitis is. The compliance with the rest of the information was as follows: investigations done to confirm the diagnosis, 60%; treatment of acute pancreatitis, 40%; potential complications, 33%; impact of smoking and alcohol, 73%; verbal information, 73%; and explanation with a diagram, 6%. None of the 30 patients were provided with a written information leaflet. It was further revealed that no patient information leaflet (PIL) was available on acute pancreatitis, despite being a common surgical presentation in our hospital. We designed a PIL and distributed it to the surgical wards after getting it approved by the surgical lead.

**Figure 2:**
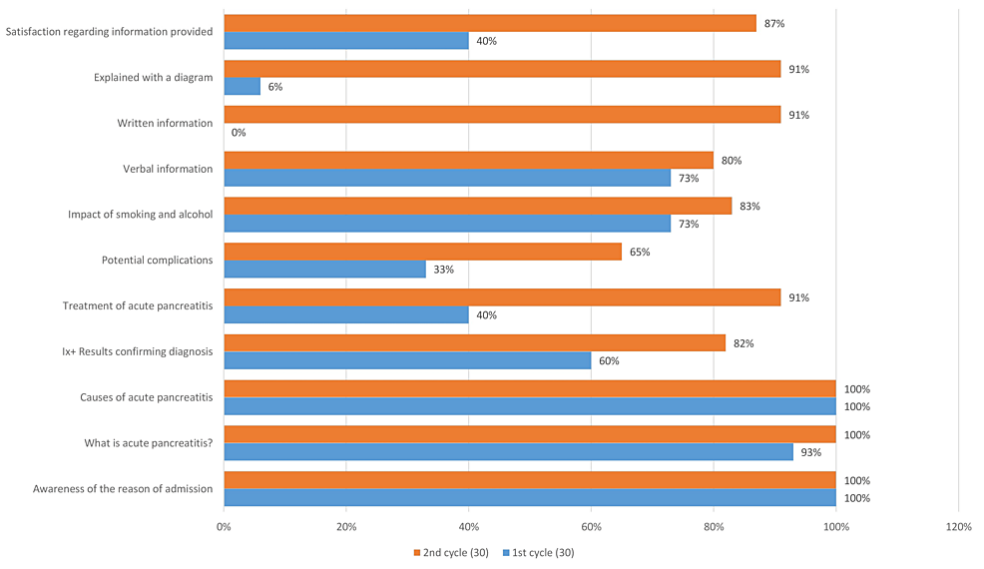
Comparison of results of the first and second audit cycles (post-intervention)

The results were presented in the audit meeting, and the junior doctors (foundation doctors and registrars) were emailed with the audit results and encouraged to provide the information along with the leaflet to the patients. The ward clerks in the surgical wards were also provided with the leaflet to be kept in the wards and encouraged to provide it to the patients with acute pancreatitis. The second half of the audit was then conducted two months after implementing the interventions to assess compliance with the GMC and NICE good medical practice. The results from both audits have been summarized in the following diagram (Figure 2).

## Discussion

This audit cycle examined one of the most important pillars of GMC good medical practice and NICE guidelines regarding the right to information of patients admitted to our hospital with acute pancreatitis, which is one of the common surgical presentations in the hospital. The vast majority of European research conducted over the past five decades have revealed that the incidence of acute pancreatitis is increasing by about 3.4% every year [13].

With this much rise in the incidence, it is now becoming increasingly imperative due to medicolegal reasons to ensure that patients are aware of the medical conditions they are admitted with and are being treated for. Moreover, patient satisfaction is a crucial and frequently used metric for assessing the quality of healthcare service. A simple intervention with the help of a leaflet in our case not only raised awareness among the patients but also increased satisfaction by more than 100%. This has also been evidenced in one of the studies by Gibbs et al. [14], which examined the impact of PILs containing information on mediation and revealed that patients who received the leaflets were much more satisfied with their care than patients who did not.

The study by Dennis et al. [15] determines that educating patients how to manage their chronic conditions on their own can enhance health outcomes and quality of life indicators, and a study by Hocking et al. [16] suggests that the ability of a patient to self-manage a chronic ailment depends on that patient’s awareness of the problem. Similarly, educating patients on the causes of acute conditions such as pancreatitis, such as alcohol and gallstones, can help modify lifestyles, potentially reducing the incidence of the condition. A number of studies have suggested that patient information materials do improve patient health outcomes if used and appreciated by patients [17]. This is also evident in one of the systematic reviews conducted by de Bont et al. [18], which examined the impact of PILs on the frequency of antibiotic prescriptions and found a similar result. It was discovered that the distribution of leaflets on common diseases resulted in decreased rates of reconsultations and fewer prescriptions for antibiotics.

Studies conducted by Weiss et al. [19] and Graham et al. [20] also recommend written materials in a patient-friendly manner to be provided to the patients to ensure increased understanding and clarity, which is in line with the GMC good medical practice guidelines clause number 32: “You must give patients the information they want or need to know in a way they can understand.” Patients who are better informed tend have a better understanding of what to expect and know what precaution they must take, which can result in better outcomes, as has also been suggested by Zirwas et al. [21], outlining the satisfaction and adherence to the treatment achieved by effective education with the help of verbal, written, and various other means of providing information.

## Conclusions

This study shows a significant improvement in all factors assessed in patients admitted with acute pancreatitis, especially doubling the overall satisfaction of the patients with the information provided to them after interventions in the form of patient information leaflets. This study concludes that patients should be provided with all the necessary information according to their right to information as per GMC best practice and NICE and Royal College of Nursing guidelines. Patient satisfaction is a crucial and frequently used metric for assessing the quality of healthcare service. Effective education improves adherence to therapy, which leads to better outcomes and promotes patient satisfaction. Patient health outcomes and satisfaction can best be achieved by providing them with a patient information leaflet. Patient information leaflets have been crucial in enabling patients to weigh their options and comprehend what they might experience throughout the treatment, especially when doctors have limited time to discuss them with the patient.
